# Gulls as potential sentinels for urban litter: combining nest and GPS-tracking information

**DOI:** 10.1007/s10661-023-11133-9

**Published:** 2023-03-29

**Authors:** Eve Galimany, Joan Navarro, Ilaria Martino, Raül Aymí, Pablo Cermeño, Tomas Montalvo

**Affiliations:** 1 Institut de Ciències del Mar (ICM), CSIC, Passeig Marítim de la Barceloneta 37-49, 08003 Barcelona, Spain; 2 Institut Català d’Ornitologia, Museu de Ciències Naturals de Barcelona, Pl. Leonardo da Vinci, 4-5, a, 08019 Barcelona, Spain; 3 Research and Conservation Department, Barcelona Zoo Foundation, Parc de La Ciutadella, 08003 Barcelona, Spain; 4 Servei de Vigilància I Control de Plagues Urbanes, Agencia de Salud Pública de Barcelona, Pl. Lesseps, 1, 08023 Barcelona, Spain

**Keywords:** Coastal city, One health, Plastic, Sentinel species, Urban ecology, Waste management

## Abstract

The production of urban waste has increased in the past decades leading to its mishandling. The effects on public health, economy, and wildlife that waste mismanagement can have are forcing governments to increase their efforts in detecting and mitigating the presence of waste. Identifying and monitoring sentinel species to assess the presence of urban litter could be a cost-effective option. Thus, analyzing the nest composition of yellow-legged gulls from an urban population inhabiting a very high populated city (Barcelona, Spain), and combining this information with accurate GPS tracking data, provides a potential tool to monitor the presence of marine and terrestrial litter over time. The results revealed the highest presence of debris in the nests of a seabird ever recorded. All the nests examined contained anthropogenic waste, with plastic items present in all of them. Crossing the nest composition with GPS tracking movements confirmed that the waste to build the nests was collected in the urban area and not in other environments surrounding the city. Then, the nest waste composition may be a good indicator of waste mismanagement and advise the municipalities to improve waste management and recycling strategies for the different types of litter. Using gulls breeding in cities as sentinel species and, in particular, the study of their nest composition, may provide essential data to decision-making stakeholders to adopt a One Health approach and help improve not only the environment’s health but also the health of those who live in it.

**Supplementary Information:**

The online version contains supplementary material available at 10.1007/s10661-023-11133-9.

## Introduction

Coastal urban areas have increasingly grown in the last decades, and half of the population live in cities with over 100,000 people located within 100 km from the coast (Barragán & de Andrés, 2015). Accordingly, the production of municipal waste has been progressively increasing during the last decades, and it is predicted to double by 2025 (Hoornweg & Bhada-Tata, 2012). The correlation between municipal waste volume and resident population is linear; for each unit increase in population, the quantity of waste increases with an exponent of 1.06 (Wowrzeczka, 2021). As a result, the amount of urban waste grows faster than the number of inhabitants. This fact leads towards the generation of mismanaged urban waste, which is composed of urban litter (incorrectly disposed waste), and inadequately contained waste, which can be spread via wind and runoff from terrestrial to aquatic habitats (Ballatore et al., 2022; Lebreton & Andrady, 2019). Solid waste mismanagement is worsened by unsustainable practices that result in environmental contamination and the spread of diseases (Ferronato & Torretta, 2019). Consequently, this issue can have significant detrimental effects on public health, economy, and urban wildlife (Ballatore et al., 2022). To prevent these effects, governments are putting particular emphasis on detecting urban waste; therefore, identifying the use of sentinel species to monitor the presence of urban litter could be a cost-effective option (Acampora et al., 2016; Multisanti et al., 2022).

Sentinel organisms are a form of health indicators that serve as proxies for environment health signaling warnings, at different levels, about potential impacts on a specific ecosystem (Tabor & Alonso Aguirre, 2004). Several species have been identified to trace environmental pollution, including both land and aquatic ecosystems, and different types of pollution. For example, the osprey (*Pandion haliaetus*) has been used as sentinel species in freshwater habitats such as rivers, lakes, reservoirs, and estuaries to inform the effects and the presence of different environmental pollutants (Grove et al., 2009). Similarly, the loggerhead turtle (*Caretta caretta*) has been proposed as target indicator to monitor the impact of marine litter in aquatic ecosystems (Matiddi et al., 2017). To be considered a sentinel organism, it must meet particular requirements, including a well-known biology and natural history, a wide distribution, and availability in sufficient numbers, among others (Basu et al., 2007). Thus, in coastal cities, urban-dwelling wildlife can be used as litter sentinels. Among them, opportunistic large gulls could be good sentinel candidates to be used as tracers of marine and terrestrial litter due to their wide plastic behavior, foraging in both marine and urban habitats (Coccon et al., 2022; Gimeno et al., 2022; Langley et al., 2023; Méndez et al., 2020; Navarro et al., 2016; Spelt et al., 2019).

The availability of diverse food resources within cities has led gull populations to develop worldwide (Belant, 1997; Martín-Vélez et al., 2022; Meléndez-Arteaga et al., 2022; Spelt et al., 2019). As coastal and marine predators, gulls also obtain resources from fishing vessels, exploiting their discard fraction (Bartumeus et al., 2010; Gimeno et al., 2022; Pais de Faria et al., 2021). The increase of uncontrolled waste, including marine litter, in seaside cities, provides abundant anthropogenic material for gulls to be incorporated in their nests (Lato et al., 2021; Lopes et al., 2020; Thompson et al., 2020; Yaghmour & Al Marashda, 2020). This material can be quantifiable by researchers (Ryan, 2020; Tavares et al., 2020) which, along with the gulls’ behavior and the possibility to investigate the habitat used by urban gulls using accurate GPS instruments (Martín-Vélez et al., 2022; Rock et al., 2016; Spelt et al., 2019), allows a better understanding of the levels of mismanaged waste within their inhabiting ecosystems. Moreover, it may also allow inferring in the origin of the waste present in the nests.

Here, we aim to demonstrate how the analysis of the nest composition in combination with accurate GPS tracking information of an urban gull population could be used to monitor the presence of marine and terrestrial litter, not only on an ad hoc basis, but also over time to envisage trends or changes. In particular, we examined both the presence of anthropogenic material within the nests and the main habitats used by the urban population of yellow-legged gulls (*Larus michahellis*) inhabiting the crowded city of Barcelona (NE Spain). All this information highlighted the amount and type of litter in coastal cities, along with the potential habitats where this litter was collected by the urban gulls, supporting the value to use gulls as sentinel species to track mismanaged waste in cities.

## Materials and methods

### Fieldwork procedures

**Fig. 1:**
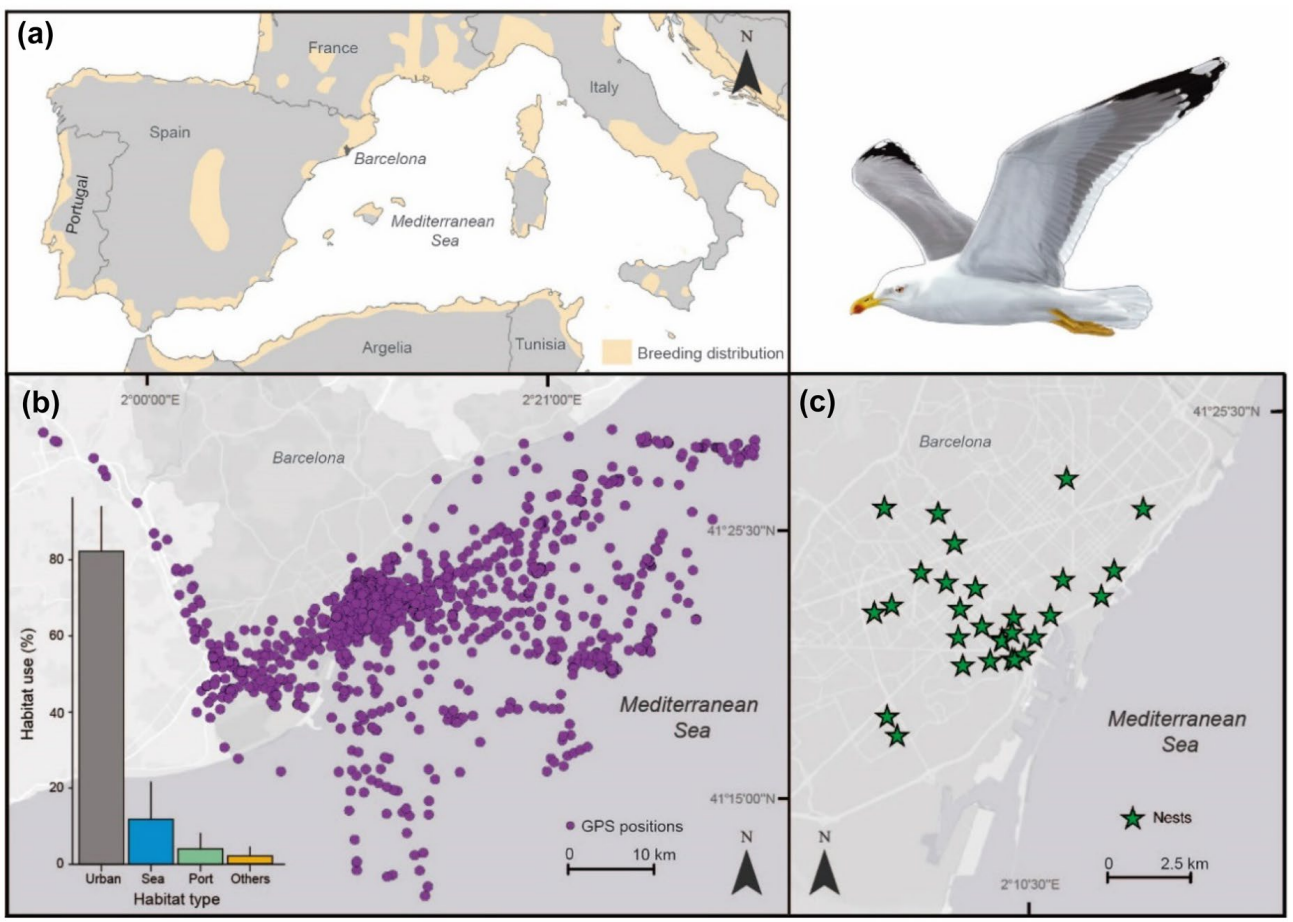
(**a**) Location of Barcelona city within the western Mediterranean Basin. (**b**) GPS positions and habitat use of 20 GPS-tracked yellow-legged gulls during 2021 breeding season in Barcelona. (**c**) Distribution of the 30 nests sampled in Barcelona during the 2021 breeding season to examine the presence of marine and terrestrial litter. Gull drawing by Martí Franch

This study was developed during May and June 2021 in Barcelona (NE Iberian Peninsula, Spain, Fig. 1(a)), one of the largest cities of Europe (1.6 million of people; Eurostat). The urban population of yellow-legged gull of Barcelona has been estimated in ~ 500 pairs (Anton et al., 2017). The urban ecosystem of Barcelona surrounded by a fishing port, recreational harbors, urban waste installations, agricultural areas, and rivers provide easy-to-catch food to opportunistic gulls (Carmona et al., 2021; Méndez et al., 2020).

To determine the presence of litter in the nests, we examined the composition of 30 nests of yellow-legged gull randomly collected throughout the city of Barcelona at the beginning of the incubation period (Fig. 1(c)). All the nests were collected by the Public Health Agency of Barcelona (following the Legislative Decree 2/2008, 15 April, DOGC). To investigate the spatial movements of breeding adults, 9 adult yellow-legged gulls were instrumented with GPS units (CatLog, Perthold Engineering LLC; 16 g of weight, corresponding to less than the 3% of the weight of yellow-legged gulls) that recorded the GPS position of each bird every 5 min, 24 h (Fig. 1(b)). The gulls were captured at the beginning of the breeding period in a trap (baited with fish) in a metropolitan park of Barcelona, and GPS devices were attached using a conventional Teflon harness (Thaxter et al., 2014). All these individuals had the presence of irrigated incubation patch, indicating that they were active breeders.

### Nest litter quantification

All the litter from the nests was classified based on the Master List category of items from the European Marine Strategy Framework Directive (Galgani et al., 2013). In general, the list classifies the items according to material type, i.e., plastic, textile, paper, rubber, metal, glass and ceramics, processed wood, and others. However, after the analysis of the items found, the categories reported are as follows: plastic, paper, metal, and others (see Table 1). The other categories from the Master List were not present or were very scarce, thus were included in the category others. From each of these types of items, there are sub-categories, such a hygienic, which we considered important to report because it includes personal hygiene and health-related items such as disposable wet wipes or single-use gloves. The material studied did not include pellets that might have been regurgitated by the gulls, and only those items nicely packed in the nest were identified and quantified. All litter items from the nests were counted and weighed (in g), and three metrics were calculated: %FO (frequency of occurrence of each type of litter in relation to the total number of nests analyzed), %N (contribution by number of each type of litter in relation to the total number detected), and %W (contribution by weight of each type of litter in relation to the total weight of the nest). ANOVA and SNK post hoc tests were used to test for differences in weight among the different types of litter present in the nests. IBM SPSS v.28 was used to run the statistical tests.

**Table 1:** List of the different types of litter found in the yellow-legged gull’s nests from Barcelona in 2021 indicating the percentage of weight (% W) of each litter and the potential habitat where each type of litter could be present (X)

		Habitat						
Type of litter	%W	Urban	Port	Dump	Agriculture	Freshwater	Sea	Beach
Plastic	**68.83**	**X**	**X**	**X**	**X**	**X**	**X**	**X**
Cigarette buds	0.29	X	X	X				X
Bags (shopping, food, freezer)	0.74	X	X	X			X	
Lids	0.01	X				X	X	X
Packaging related to candy	0.00	X		X		X	X	X
Food packaging (fast food) and cosmetics	0.11	X		X				
Packaging (cable ties, tapes, …)	3.01	X	X	X	X			
Ropes/Strings	4.66	X	X	X	X	X	X	
Foam	1.42	X	X	X		X	X	
Can holders	0.05	X		X				X
Plastic pieces 0–2.5 cm (pellets)	1.76	X	X	X	X	X	X	X
Plastic pieces 2.5 cm-50 cm	7.67	X		X				X
Other plastic objects (pens, lighters, …)	0.30	X		X				X
Polystyrene	0.00	X	X	X		X	X	X
All insulation materials	46.96	X		X				
Clothespin	0.19	X						
Medical stuff	0.18	X		X		X	X	X
Plastic net (gardening)	1.10	X			X			
Paper/cardboard	**5.79**	**X**	**X**	**X**	**X**	**X**	**X**	**X**
Paper napkins, table cloths	1.02	X		X				
Boxes and box pieces	2.20	X	X	X	X			
Drink cardboards	0.06	X		X				X
Tobacco packs	0.12	X		X				X
Paper pieces	2.87	X		X		X	X	
Wood (processed)	**0.65**	**X**	**X**	**X**	**X**	**X**	**X**	**X**
Corks	0.02			X				
Sticks	0.11	X		X	X			X
Other objects and pieces < 50 cm	0.50	X	X	X	X	X	X	X
Metal	**2.96**	**X**	**X**	**X**	**X**	**X**	**X**	**X**
Lids and covers for bottles and cans	0.04	X		X				X
Aluminum foil	0.32	X		X		X	X	X
Other objects and pieces < 50 cm	2.63	X	X	X	X			X
Glass	**0.05**	**X**	**X**	**X**	**X**	**X**	**X**	**X**
Bottles and containers	0.02	X		X				X
Other objects and pieces	0.03	X	X	X				X
Hygienic waste	**0.78**	**X**	**X**	**X**	**X**	**X**	**X**	**X**
Ear buds	0.00	X		X				X
Wet wipes	0.76	X	X	X				X
Others	**20.95**	**X**	**X**	**X**	**X**	**X**	**X**	**X**
Rubber (balloons, balls, strings, valves, …)	0.07	X	X	X	X	X	X	X
Other textile	14.97	X		X				X
Construction materials (bolts, nails, …)	1.29	X	X	X	X			
Other objects and ceramic pieces	0.10	X		X				
Brushes (mixed materials)	4.37	X	X	X				

Bold indicates % W and presence of items for the main categories

### Spatial movement of gulls

To characterize the habitat utilized by the GPS-tracked yellow-legged gulls, we estimated the % of use of each habitat type by each GPS-tracked individual. Specifically, we calculated the % between the number of positions in each type of habitat divided by the total GPS fixes for each GPS-tracked gull. Habitat type was determined by overlapping the GPS with land-cover data (Urban Atlas, European Environment Agency; Copernicus Land Monitoring Services 2016; < 5 m resolution), aggregated into 7 categories: urban, beach, port, sea, freshwater, landfill, and agriculture. Each of these habitats could be the source of the different types of litter present in the nests (Table 1). To perform spatial analysis, we used QGIS 3.10. A Coruña (QGIS Development T 2019).

## Results and discussion

### Anthropogenic waste in the nests

**Fig. 2:**
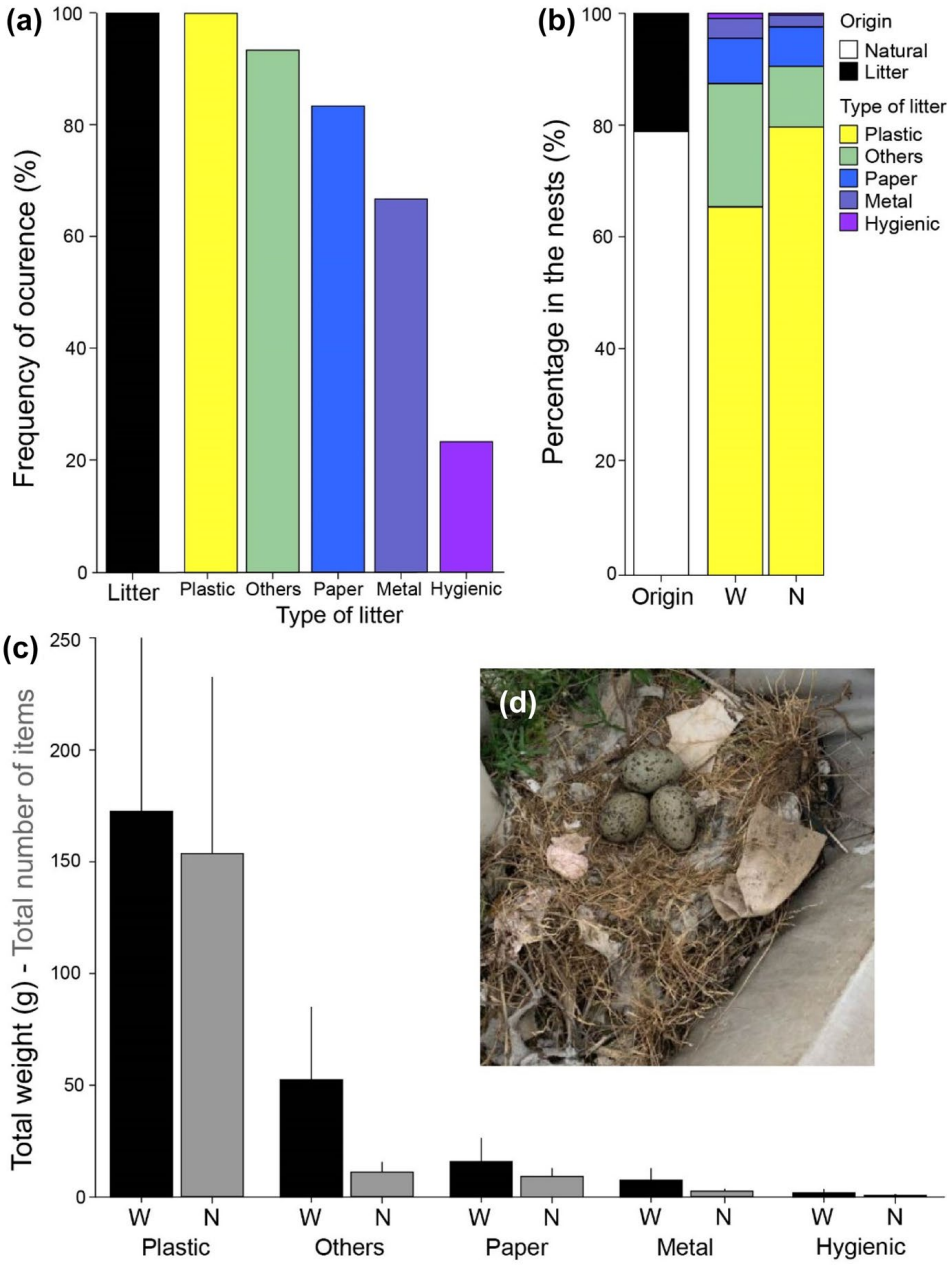
**a** Frequency of occurrence of litter and the different types of litter in the nests of yellow-legged gulls from Barcelona. **b** Percentage by the origin of the items (natural or litter), and by the type of litter present in the nests of the yellow-legged gulls from Barcelona by weight (*W*) and number (*N*). **c** Mean and 95% confidence interval of the presence of each type of litter in the nests of yellow-legged gulls from Barcelona by weight (*W*) and number (*N*). **d** Picture showing a yellow-legged gull nest located on one roof of Barcelona (picture by Tomás Montalvo)

Recent studies have described the presence of anthropogenic waste in the nest of yellow-legged gulls and similar gulls (Battisti, 2020; Lato et al., 2021; Lopes et al., 2020; Pon & Pereyra, 2021; Thompson et al., 2020). These studies were mostly conducted in natural environments, with less availability of anthropogenic waste in the surroundings of the colonies, whereas we centered the study in an urban population inhabiting a very high–populated city. In particular, all the nests examined contained anthropogenic waste (Fig. 2a), accounting for the 21% of the total weight of the nests (Fig. 2b; Table 1). Regarding the type of waste found, plastic items were present in all of them (frequency of occurrence = 100%, Fig. 2a), followed by the categories others (93%), paper (83%), metal (66%), and hygienic waste (23%) (Fig. 2a). Statistically, plastic also showed the highest values in weight (ANOVA tests, *F*_*4,* 29_ = 14.14, *p* < 0.001) and number of items (*F*_*4,* 29_ = 17.87, *p* < 0.001) in comparison with the other type of waste, which did not differ between them (*p* > 0.05) (Fig. 2c, Table 1). The present study, then, reports the highest presence of debris in the nests of a seabird so far.

From the items analyzed, it seems that the gulls probably collected some of the waste directly from the buildings’ roofs. The most evident hint was the presence of clothes (socks or panties) or clothespins in the nests (Table 1; Fig. S1) which the yellow-legged gulls most likely found in the clothing lines area of the buildings. Another example is the presence of a type of insulation material used on the roofs of the buildings (Fig. S1). A similar material was also found in urban yellow-legged gull nests in Porto, Portugal (Lopes et al., 2020). In fact, this is the main type of plastic found in the nests, accounting for the 46% of the weight (Table 1). As this type of waste is not considered an anthropogenic-origin litter per se, but an escape from the urban waste management, we suggest improving the fixation of this material to avoid the release of a high amount of this type of non-woven fabric, a type of plastic material, to the environment.

The presence of plastic, paper, metal, or glass in the nests mirrors the occurrence of this type of waste in the environment (Li et al., 2016), and thus, it is a sign of an incorrect municipal waste management. In this line, and with the final aim to prevent the harmful consequences of anthropogenic waste, the Barcelona City Council, similarly to initiatives from other European countries, has started a recycling improvement plan, with door-to-door collection campaigns aiming to improve the recycling figures that each citizen generates home (Barcelona City Hall waste management plan). This strategy has been proven very effective to increase household recycling and might help decrease waste mismanagement (Halvorsen, 2012), which may be traced with the composition of the gulls nests (Burger & Gochfeld, 2004; Lavers et al., 2013; Tavares et al., 2020).

### Habitat used by urban gulls

The analysis of the nest composition is an essential aspect to detect the presence of waste, but the possibility to identify the habitats used by yellow-legged gulls during their foraging movements is also important to relate where the waste was collected (Table 1). Here, by using GPS tracking data from nine breeding gulls, we were able to identify the main habitats covered by them and, thus, the potential origin of the litter found in the nests (Table 1). GPS-tracking data revealed that the main habitat used by adult yellow-legged gulls in Barcelona was the urban environment (mean ± standard deviation of the GPS positions = 82.21 ± 15.64%), followed by sea (11.81 ± 12.82%), port (4.03 ± 5.93%), agriculture (1.09 ± 3.23%), freshwater (0.73 ± 2.18%), dump (0.02 ± 0.07%), and beach (0.1 ± 0.28%) (Fig. 1b). Crossing the habitat use data with the waste found in the nests, this study suggests that almost all anthropogenic waste present in the nests was collected in the urban environment. Although we did not analyze the nests of the GPS-tracked gulls, this finding reinforces the idea that nesting material is collected locally by gulls (Thompson et al., 2020). Thus, long-term determination of the litter present in gull nests could be suitable to help in monitoring the efficiency of urban management and the amount of human-related waste present in the habitats where gulls are foraging (Burger & Gochfeld, 2004; Grant et al., 2018; Tavares et al., 2020).

### Gulls as sentinel species

The use of gulls as a sentinel species for environmental health might not be a new concept (Burger & Gochfeld, 2004; Lavers et al., 2013). The analysis of the content of their nests, in combination with tracking information of breeding individuals, could open a new scenario to monitor the presence of anthropogenic waste in the environment (O’Hanlon et al., 2017). In particular, the current results open new possibilities to help detect and correct management of municipal waste. Areas such as dumpsters, constructions sites, gardens and parks, or beaches may be potential main sources of abandoned solid waste, where the local administration may need to focus to improve waste management strategies. The results also deepen in the knowledge of the impact that these residues can create in the environment. This approach complies with the “One Health” strategy that interrelates human, animal, and environmental health (Ewbank et al., 2021; Mackenzie & Jeggo, 2019). Measuring the variables that contribute to urban health is a challenge to promote healthier and more equitable cities. Seabirds can act, then, as sentinels of natural and anthropogenic changes in the health of the marine and terrestrial ecosystems (Thibault et al., 2019). Thus, there are compelling reasons to develop new approaches to help improve the detection, prevention, and monitoring of the parameters that affect global health. One of the objectives of the World Health Organization (WHO) is to have a Single Health Observatory to bring together data from different sources. That is why, using a species widely distributed in cities such as the yellow-legged gull, especially for the role it plays in terms of habitat use and nest composition, can provide information on the state of health of the environment. In this sense, further studies can also provide knowledge on animal health and the possible impacts on human welfare that may result. However, because the nest construction only occurs for a small period of time of the breeding season (i.e., 1 month), assembling these data with other types of information, such as non-breeding season tracking data and pellet analysis, for instance, could improve the understanding of the health state of the environment throughout the year. The integration, interpretation, and evaluation of the information obtained from monitoring the nests and other type of data should provide clues to the managers (ex. urban clean-up or recycling) on how citizens manage their waste. That would allow assistance in decision-making to adopt a One Health approach and thus achieving the goal to improve health and well-being, especially in overcrowded coastal cities.

## Conclusions

The yellow-legged gulls from an urban population inhabiting a very high–populated city (Barcelona, Spain) had the highest presence of debris in the nests of a seabird so far. All the nests examined contained anthropogenic waste, accounting for the 21% of the total weight of the nests, with plastic items being present in all of them. When analyzing the composition combined with tracks, it was evidenced that the waste to build the nests was collected in the urban area and not in other environments surrounding the city. Then, the nest waste composition may be a good indicator of waste mismanagement and an advice to the municipalities to improve waste management and recycling strategies for the different types of litter. Proposing yellow-legged gulls as sentinel species, and in particular the study of their nest composition, may provide essential data to decision-making stakeholders to adopt a One Health approach and help improve not only the environment’s health but also the health for those who live in it.

## Supplementary Information

Below is the link to the electronic supplementary material.

Supplementary file1 (DOCX 433 KB)

## Data Availability

Data will be made available on request.
