# Supplementary material for: Gulls as potential sentinels for urban litter: combining nest and GPS-tracking information
Source: Environ Monit Assess. 2023 Mar 29;195(4):521. doi: 10.1007/s10661-023-11133-9 (PMC10060334; doi:10.1007/s10661-023-11133-9)
Supplement: Supplementary file 1 — Supplementary file1 (DOCX 433 KB) [file 10661_2023_11133_MOESM1_ESM.docx]

**SUPPORTING MATERIAL**

**Gulls as potential sentinels for urban litter: combining nest and GPS-tracking information**

Eve Galimany^1,*^, Joan Navarro^1^ , Ilaria Martino^1^, Raül Aymí^2^, Pablo Cermeño^3^ and Tomas Montalvo^4^

^1^ Institut de Ciències del Mar (ICM), CSIC, Passeig Marítim de la Barceloneta 37-49 08003 Barcelona, Spain

^2^ Institut Català d’Ornitologia, Museu de Ciències Naturals de Barcelona, Pl. Leonardo da Vinci, 4-5, a, Barcelona, 08019, Spain

^3^Research and Conservation Department. Barcelona Zoo Foundation, Parc de la Ciutadella, 08003 Barcelona, Spain

^4^ Servei de Vigilància i Control de Plagues Urbanes, Agencia de Salud Pública de Barcelona, Pl. Lesseps, 1, 08023 Barcelona, Spain

*Corresponding author: [galimany@icm.csic.es](mailto:galimany@icm.csic.es)

**Figure S1.** (a-b) Examples of marine and terrestrial litter found in the yellow-legged gull nests of Barcelona in 2021. (c-e) Insulation material found in the yellow-legged gull nests of Barcelona in 2021: insulation material in a nest (c), on the roof of a building (d), and separated in the laboratory (e). Pictures were taken by Ilaria Martino and Joan Navarro.

**
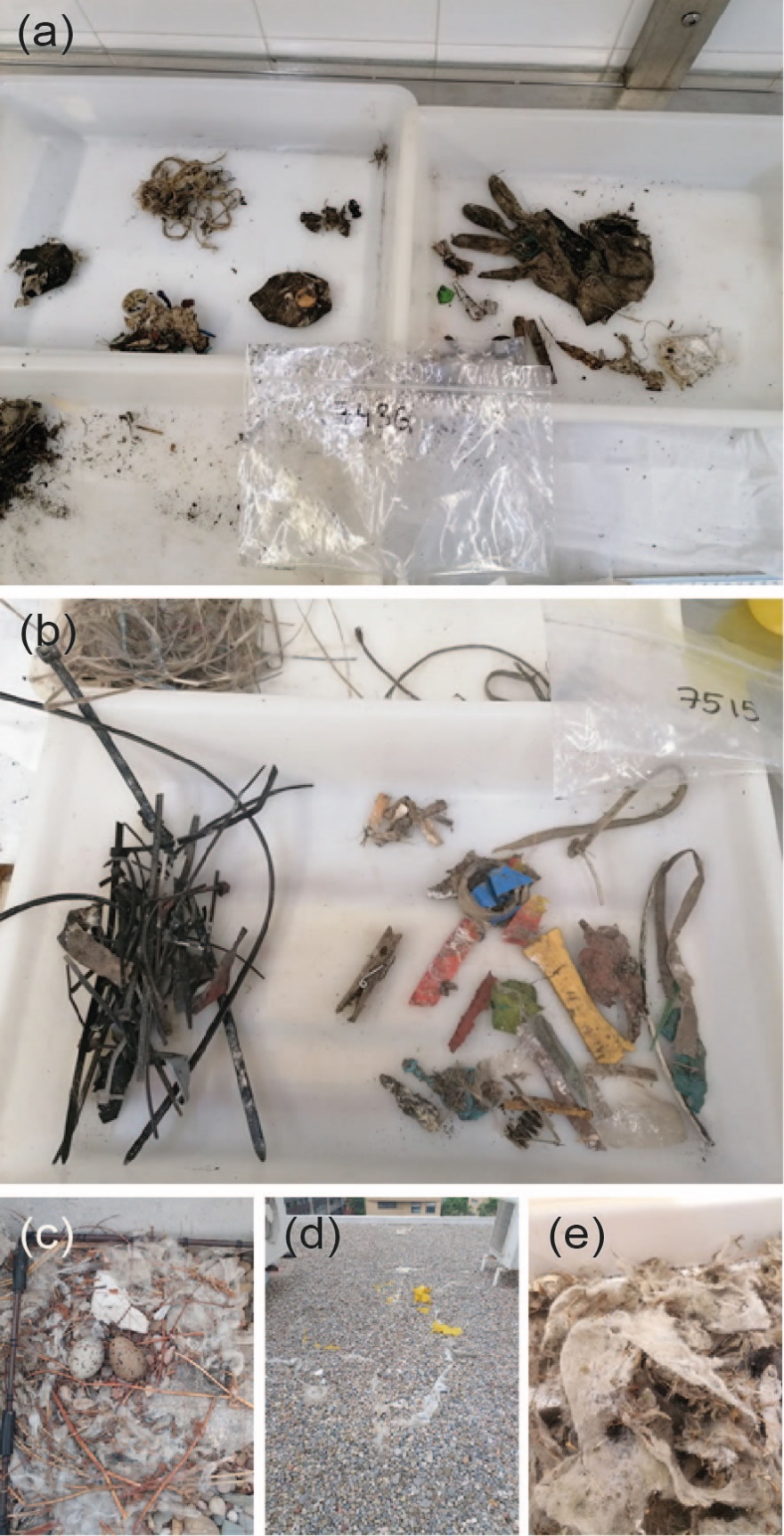
**
